# [6]-Gingerol, from *Zingiber officinale*, potentiates GLP-1 mediated glucose-stimulated insulin secretion pathway in pancreatic β-cells and increases RAB8/RAB10-regulated membrane presentation of GLUT4 transporters in skeletal muscle to improve hyperglycemia in Lepr^db/db^ type 2 diabetic mice

**DOI:** 10.1186/s12906-017-1903-0

**Published:** 2017-08-09

**Authors:** Mehdi Bin Samad, Md. Nurul Absar Bin Mohsin, Bodiul Alam Razu, Mohammad Tashnim Hossain, Sinayat Mahzabeen, Naziat Unnoor, Ishrat Aklima Muna, Farjana Akhter, Ashraf Ul Kabir, J. M. A. Hannan

**Affiliations:** 1 Department of Pharmaceutical Sciences, University of Nebraska Medical Center, Omaha, NE USA; 2 Department of Pharmaceutical Sciences, North South University, Dhaka, Bangladesh; 3 Department of Pharmacy, BRAC University, Dhaka, Bangladesh; 4 Seoul National University, Seoul, South Korea; 5 Washington University School of Medicine in St. Louis, St. Louis, MO USA; 6 Department of Pharmacy, East West University, Dhaka, Bangladesh

**Keywords:** [6]-Gingerol, Lepr^db/db^ mice, Type 2 diabetes, GLP-1, Rab27a, GLUT4, Glycogen synthase 1, Rab8, Rab10

## Abstract

**Background:**

[6]-Gingerol, a major component of *Zingiber officinale*, was previously reported to ameliorate hyperglycemia in type 2 diabetic mice. Endocrine signaling is involved in insulin secretion and is perturbed in db/db Type-2 diabetic mice. [6]-Gingerol was reported to restore the disrupted endocrine signaling in rodents. In this current study on Lepr^db/db^ diabetic mice, we investigated the involvement of endocrine pathway in the insulin secretagogue activity of [6]-Gingerol and the mechanism(s) through which [6]-Gingerol ameliorates hyperglycemia.

**Methods:**

Lepr^db/db^ type 2 diabetic mice were orally administered a daily dose of [6]-Gingerol (200 mg/kg) for 28 days. We measured the plasma levels of different endocrine hormones in fasting and fed conditions. GLP-1 levels were modulated using pharmacological approaches, and cAMP/PKA pathway for insulin secretion was assessed by qRT-PCR and ELISA in isolated pancreatic islets. Total skeletal muscle and its membrane fractions were used to measure glycogen synthase 1 level and *Glut4* expression and protein levels.

**Results:**

4-weeks treatment of [6]-Gingerol dramatically increased glucose-stimulated insulin secretion and improved glucose tolerance. Plasma GLP-1 was found to be significantly elevated in the treated mice. Pharmacological intervention of GLP-1 levels regulated the effect of [6]-Gingerol on insulin secretion. Mechanistically, [6]-Gingerol treatment upregulated and activated cAMP, PKA, and CREB in the pancreatic islets, which are critical components of GLP-1-mediated insulin secretion pathway. [6]-Gingerol upregulated both *Rab27a* GTPase and its effector protein *Slp4-a* expression in isolated islets, which regulates the exocytosis of insulin-containing dense-core granules. [6]-Gingerol treatment improved skeletal glycogen storage by increased glycogen synthase 1 activity. Additionally, GLUT4 transporters were highly abundant in the membrane of the skeletal myocytes, which could be explained by the increased expression of *Rab8* and *Rab10* GTPases that are responsible for GLUT4 vesicle fusion to the membrane.

**Conclusions:**

Collectively, our study reports that GLP-1 mediates the insulinotropic activity of [6]-Gingerol, and [6]-Gingerol treatment facilitates glucose disposal in skeletal muscles through increased activity of glycogen synthase 1 and enhanced cell surface presentation of GLUT4 transporters.

**Electronic supplementary material:**

The online version of this article (doi:10.1186/s12906-017-1903-0) contains supplementary material, which is available to authorized users.

## Background

*Zingiber officinale* Roscoe (Zingiberaceae), known as ginger, is one of the most widely consumed spices worldwide [1, 2]. Ginger has long been used in complementary and alternative medicine preparations for the treatment of different diseases, such as vomiting, pain, indigestion, and cold-related symptoms [3]. [6]-Gingerol ((S)-5-hydroxy-1-(4-hydroxy-3methoxyphenol)-3-decanone) is an aromatic polyphenol that is a major constituent of ginger. Previous studies on rodents reported antioxidant [3], analgesic [4, 5], anti-inflammatory [6], and anti-tumor and pro-apoptotic [7–9] properties of [6]-Gingerol. Interestingly, a recent study reported that [6]-Gingerol also has potent insulin secreting, anti-hyperglycemic, lipid lowering, and anti-oxidant properties in a Lepr^db/db^ type 2 diabetic mouse model [10], all of which are essential hallmarks of an effective anti-diabetic agent. From a mechanistic point of view, it has been demonstrated in an obese Lepr^db/db^ type 2 diabetic mouse model that [6]-Gingerol regulates hepatic gene expression of enzymes related to glucose metabolism [11]. [6]-Gingerol was also found to improve the serum lipid profile and hepatic expression of related metabolic genes in a high-fat fed rat model, which eventually alleviated diabetes-related complications [12]. However, the underlying mechanism of action of [6]-Gingerol-induced insulin secretion for ameliorating hyperglycemia are yet to be fully understood. In this study, we aimed to characterize the mechanism(s) through which [6]-Gingerol induces insulin secretion and exerts its antihyperglycemic potential. Understanding the mechanism would enable us to design [6]-Gingerol-based novel anti-hyperglycemic agents.

Insulin resistance and lack of insulin secretion due to pancreatic β-cell failure are among the leading causes of type 2 diabetes [13]. Endocrine hormones are involved in nutrient-stimulated insulin secretion, also known as the incretin effect [14]. [6]-Gingerol was found to restore the disrupted endocrine signaling in the testes and the epididymis of rats [15]. [6]-Gingerol was also reported to play a major role in glucose metabolism via regulating the intracellular trafficking of glucose transporter in skeletal muscle cells [16]. Intracellular vesicular transport is crucial for the second-phase of the biphasic insulin release in response to glucose, which is essential for maintaining glucose homeostasis [17]. Our current study was designed to test the hypothesis that [6]-Gingerol regulates endocrine signaling and intracellular trafficking to increase glucose-stimulated insulin secretion and peripheral glucose utilization, which, in turn, improve the hyperglycemic condition in type 2 diabetes. Here, we report that [6]-Gingerol stimulates GLP-1 mediated insulin secretion pathway and upregulates *Rab27a/Slp4-a* that control insulin granule exocytosis in pancreatic β-cells, and facilitates glucose disposal in skeletal muscle by up-regulating glycogen synthase 1 and by increasing GLUT4 membrane presentation.

## Methods

### Chemicals

All the chemicals and reagents including [6]-Gingerol were purchased from Sigma-Aldrich, USA, unless stated otherwise. Saxagliptin was purchased from a commercial pharmacy retailer in Dhaka, Bangladesh (brand name: Onglyza; ASTRA ZENECA).

### Animal handling

Type 2 diabetic mice (Lepr^db/db^) were procured from Harlan Laboratories (USA) and were raised in the animal house of the Department of Pharmaceutical Sciences, North South University. The mice weighed about 25 ± 2 g. All test animals were kept in the North South University Animal Facility at an ambient temperature of 22 ± 5 °C and a humidity of 50–70%. 12 h day-night cycle was maintained for natural circadian rhythm. Standard pellets and filtered drinking water were made available to the test animals, ad libitum, throughout the experiment, apart from the period of fasting, when only water was provided. Animals undergoing fasting were placed in grilled bottomed cages, with no bedding, to prevent coprophagy. The mice were tagged with an I.D. number, which was fed into a computer program. The program allocated ten mice to each group, at random. A trained technician was responsible for administering the test compounds to the animals. To avoid any bias, the groups were randomly assigned numbers and the investigators were unaware of the medication status of the animals till data analysis was completed. A total of 110 mice were used in the study (details are given in appropriate individual methods and figure legends sections).

### Glucose homeostasis and insulin secretion measurement

Oral glucose tolerance test (OGTT) was performed following 4-weeks of daily oral [6]-Gingerol administration (200 mg/kg in corn oil) or Glibenclamide or vehicle only (*n* = 10 mice/group, total 30 mice) [18, 10, 19], following methods described previously [20]. Briefly, after overnight fasting, animals were anesthetized with an intraperitoneal injection (100 mg/kg) of pentobarbitone sodium (Therapon, Burwood, Victoria, Australia), and a Silastic catheter filled with heparinized saline (20 U/ml) was inserted into the left carotid artery. The mouse underwent a tracheotomy to facilitate breathing. A bolus of glucose (2 g/kg) was delivered into the stomach by a gavage needle (20-gauge, 38 mm long curved, with a 21/4 mm ball end; Able Scientific, Canning Vale, Western Australia, Australia), and 200 μl of blood was sampled at 0, 30, 60, 90, and 120mins for plasma glucose and insulin analyses. The blood was immediately centrifuged and the plasma was separated and stored at −20 °C until assayed. The red blood cells were re-suspended in an equal volume of heparinized saline and re-infused into the animal via the carotid artery, prior to the collection of the next blood sample to prevent anemic shock.

Fasting blood glucose and homeostatic blood glucose levels were also measured following [6]-Gingerol treatment every week throughout the treatment period of 28 days. For fasting blood glucose level, blood samples were collected from the overnight fasted subjects of different treatment groups. For homeostatic blood glucose measurement, blood samples were collected weekly in the afternoon from the treated subjects, maintained on a standard diet, throughout the treatment period.

Blood glucose levels were analyzed by GOD-PAP method (glucose kit, Randox, UK) and plasma insulin levels were determined using Mice Insulin ELISA Kit (Crystal Chem, USA)

### Blood collection and processing

After 4-weeks of daily oral [6]-Gingerol (200 mg/kg) treatment, overnight-fasted mice were fed a bolus of glucose (2 g/kg). 1 h after the glucose administration, blood samples were collected from various groups of mice though carotid artery, plasma was separated from blood, and stored at −20 °C. Afterwards, the mice were sacrificed by cervical dislocation; their pancreas and skeletal muscles were isolated and processed for isolating islets and skeletal myocytes, respectively, as described below.

### Biochemical analysis of various plasma components

Levels of various endogenous insulin secreting or inhibiting hormones were measured from the isolated plasma. Hormones that exhibited a rise in plasma levels, in correspondence with insulin increase, were further analyzed using a plethora of positive and negative controls, pharmacological enhancers, and inhibitors or antagonists of the hormones. This approach helped us fully confirm the role of these hormones in the anti-hyperglycemic activity of [6]-Gingerol. Conversely, hormones that did not show any change in levels were not studied further. Quantification of all biochemical parameters in this section were done using Colorimetric, ELISA or EIA methods following the manufacturer’s instruction accompanying the kit. Plasma Insulin levels were measured using Ultra-sensitive mice Insulin ELISA Kit (Crystal Chem, USA). Plasma Acetylcholine levels were determined by a colorimetric Choline/Acetylcholine Quantification Kit (Abcam, USA). Plasma epinephrine was measured using Epinephrine ELISA Kit (Abnova, Taiwan). Plasma norepinephrine was assayed using the norepinephrine ELISA assay kit (Eagle Biosciences Inc. USA). Plasma GIP was assayed using the Rat/mouse GIP ELISA assay kit (Total) (EMD Millipore, USA). Plasma GLP-1 was determined by using GLP-1 EIA Kit (Sigma-Aldrich, USA). Plasma VIP was assayed using the VIP ELISA assay kit (USCN Life Sciences Inc., China). Plasma PACAP was assayed using the mouse PACAP ELISA assay kit (MyBioSourceInc, USA). Plasma IFG-1 was assayed using the mouse IGF-1 ELISA assay kit (Sigma-Aldrich, USA). Plasma Pancreatic Polypeptide was assayed using the mouse Pancreatic Polypeptide ELISA assay kit (MyBioSourceInc, USA). Plasma Somatostatin was assayed using the Somatostatin EIA assay kit (Phoenix Pharmaceutical Inc. USA). Plasma DPP-IV level was measured using Mouse DPPIV ELISA kit (Sigma-Aldrich, USA). Plasma DPP4 activity was determined by the cleavage rate of 7-amino-4-methylcoumarin (AMC) from the synthetic substrate H-glycyl-prolyl-AMC (Gly-Pro AMC; Sigma), as described previously [21].

### Pharmacological modulation of GLP-1

To further confirm the role of GLP-1 in [6]-Gingerol augmented glucose-induced insulin release, we employed Exendin 9-39 (Ext9) (Sigma-Aldrich, USA), a potent GLP-1 receptor antagonist, at a dose 300 pmol/kg/min. Ext9 was administered through a femoral vein catheter continuously for 1 h, while the animal subjects were kept under sodium pentobarbital anesthesia. Saxagliptin (SxLn), an inhibitor of dipeptidyl peptidase-4 (DPP-4), was administered 4 h before the glucose load, orally at a dose of 10 μmol/.

### Mouse pancreatic islet isolation and preparation

Mouse pancreatic islets were isolated by collagenase digestion as previously described [22]. Briefly, the [6]-Gingerol treated and control mice were fully anesthetized and sacrificed by cervical dislocation. The pancreas was distended by injecting 3 mL of the digesting solution via the common bile duct. It was then removed and placed in a 50 ml vial containing 2 mL of the digesting solution. The digestion reaction was terminated by putting the vial on the ice and by adding 25 mL CaCl2 (1 mM) supplemented HBS Buffer (CAHBS). This digested pancreatic homogenate was then processed through different steps and finally suspended in the nutrient medium (glutamine-L 20 mM, penicillin 100 U/mL, streptomycin 100uL/mL, 10%FSB buffer in RPMI 1640 medium) to capture the islets. Islets were hand-picked using a wide-tipped pipette, counted and placed in 5% CO2 incubator at 37 °C.

### Analysis of GLP-1 mediated insulin secretion pathway

GLP-1 mediated insulin secretion employs a number of key signaling molecules, the most significant of which are Protein Kinase A (PKA), cyclic Adenosine Monophosphate (cAMP), and cAMP response element binding protein (CREB) [23]. In the current study, we isolated pancreatic islets from different treatment groups (500 islets per group). The islets were homogenized over ice using a glass hand-held homogenizer. The homogenates were immediately assayed for PKA activity, cAMP level, pCREB level, and Insulin levels using commercially available kits. PKA activity was determined using PKA Activity Assay kit (Arbor Assays, USA). cAMP levels were determined using cAMP Direct Immunoassay Kit (Abcam, USA). pCREB levels were measured using Phospho-CREB (S133) DuoSet IC ELISA (R&D Systems, Inc., USA). The concurrent insulin secretion in the medium was measured using Ultra-Sensitive Mouse Insulin ELISA Kit (CrystalChem, USA). Alternatively, the freshly isolated islets were processed for mRNA preparation and qRT-PCR analysis.

### RNA isolation and qRT-PCR analysis

Total RNA was collected from isolated islets using the TRIzol reagent (Invitrogen) following manufacturer’s protocol. In the case of skeletal muscle, tissues were harvested from hindlimb of the mice and preserved in RNA*later* (Invitrogen) at −20 °C; the samples were later used to prepare total mRNA. *Pka, Creb, Rab27a*, and *Slp4-a* cDNAs were synthesized from total RNA prepared from islets and *Glut4, Rab8, Rab10,* and *Rab14* cDNA were synthesized from total RNA prepared from using Super-Script first strand synthesis system (Invitrogen, CA). Different transcripts were amplified using the power SYBR GREEN PCR Master Mix (Applied Biosystems, CA), following the manufacturer’s instructions. Primers for different genes were purchased from Integrated DNA Technologies (Iowa City, IA) (Additional file 1: Table S1). Each sample was run quadruplicated along with a corresponding *B-actin* control for each sample. PCR process and data collection were done using the BioRad Mini-Opticon Real Time PCR machine and Opticon Monitor 3 Software from BioRad (Hercules, CA), respectively. Relative cDNA copy number of the target gene and *B-actin* for each sample were calculated using the delta-delta-C(t) method. Relative expression values for target genes in each treatment were normalized to the lowest relative expression level for each experiment (*n* = 5).

### In-vivo confirmation of cAMP/PKA pathway involvement

To establish the in-vivo relevance of GLP-1 triggered cAMP/PKA pathway for the insulin secretagogue activity of [6]-Gingerol, we inhibited the pathway and assessed the effect on insulin secretion. Specifically, we utilized a potent and selective inhibitor of cyclic AMP- dependent protein kinase (PKA) inhibitor H-89 (daily intraperitoneal 0.2 mg/100 g body weight for 7 days) in [6]-Gingerol treated mice (*n* = 8 mice in each group, total 32 mice) and measured plasma insulin level following an oral glucose load. Blood samples were collected 1 h after the glucose load and measured for plasma insulin level.

### Preparation of total cell membrane fraction from mice skeletal myocytes

The [6]-Gingerol treated and control animals were sacrificed 1 h after bolus glucose administration (as described above in blood collection section) by cervical dislocation; skeletal muscle from the hind limb was removed and rapidly dissected free of connective tissues. Muscle from the individual mice was placed in Tris buffer (pH 7.4, 20 mM Tris-base, 0.05 M sucrose, 0.1 mM EDTA, 5 μg/mL leupeptin, 5 μg/mL aprotinin, 1 μ/mL pepstatin, and 400 μM phenylmethanesulfonyl fluoride). 1 g of the tissue was placed in 5 mL of the Tris buffer. The total membrane fraction was prepared following methods described previously [24] with minor modifications [25]. In short, the dissected muscle tissue was finely homogenized using a glass homogenizer (five 5 s bursts at a setting of 5 and then with 10 up-and-down strokes of a Teflon pestle). Aliquots of the homogenate were immediately assayed for glycogen synthase 1 activity, muscle glycogen content, and total skeletal GLUT4 levels; the remainder was further processed to obtain the membrane fraction. The homogenate was centrifuged at 1000 g for 10mins and the supernatant was collected. The resultant pellet was re-suspended in Tris-buffer and re-homogenized using the same instrument and method, as previously described, and re-centrifuged at 1000 g for further 10mins. The resultant pellet was discarded and the second supernatant was added to the first supernatant, thoroughly hand shaken for 1 mins and centrifuged at 9000 g for 10mins. The resultant supernatant derived from this process was centrifuged at 190000 g for 1 h. This was discarded and the pellet was re-suspended which made up the total membrane fraction. The membrane was kept at −80 °C until further analysis.

### GLUT4 transporter content in total and membrane fractions of skeletal muscles

Previously prepared total cellular and membrane fractions of mouse skeletal myocytes were assayed to determine the amount of total and membrane docked GLUT4 receptors, respectively. GLUT4 transporters were quantified using ELISA kit procured from UScn Life Science Inc. (USA) following the manufacturer’s instructions.

### Glycogen synthase 1 enzyme activity

The muscle homogenate was diluted 300 times and the glycogen synthase 1 activity was assayed following a method as described previously [26]. Briefly, the reaction mixture was comprised of Tris Buffer 50 mM, MgCl_2_ 12.5 mm, EDTA 1 mM, mercaptoethanol 2.5 mM, UDP-D-glucose 0.75, and 1% glycogen. The assay was carried out in the presence of 0.1 mM and 10 mM glucose-6-phosphate. The reaction was quenched by heating in a thermostatically controlled boiling water bath for 70s. This helped to denature the proteins which were subsequently removed by centrifugation at 400 g. The supernatant was collected and assayed for UDP. This was done by reacting UDP with phosphoenolpyruvate in the presence of the enzyme pyruvate kinase. The resultant pyruvate was made to react with DPNH in presence of the enzyme lactate dehydrogenase. DPNH gradually disappeared which was spectrophotometrically followed. Results were expressed as nmol/min/mg of extract protein.

### Muscle glycogen content

Around 15 mg muscle tissue was collected from the left hind leg of the mice. 10 mg of the muscle tissue was homogenized along with 200 μl of H_2_O_2_ on the ice. The homogenate was boiled for 5mins to inactivate all enzymes. The boiled samples were centrifuged at 13000 rpm for 5mins to remove insoluble materials; the supernatant was then assayed for glycogen using a colorimetric Glycogen assay kit (Abcam, USA) as per the instruction booklet. The background glucose was measured in separate wells, before addition of the hydrolytic buffer and subtracted from the final value.

### Statistical analysis

Statistical tests were conducted using GraphPad Prism ver.7. Results were presented as mean with standard deviation for all measurements. Data from experimental groups were compared using unpaired Student’s *t*-test and the Mann–Whitney *U* test, as required. Experiments, where data were collected at several time intervals, were analyzed using repeated measures ANOVA followed by Bonferroni adjustment ensuring an error margin within ≤5%. One-way ANOVA was carried out and pair-wise comparisons were made with the control group using Dunnett’s test to maintain an acceptable error margin of 5%. A two-tailed *p*-value <0.05 was considered statistically significant.

## Results

### [6]-Gingerol treatment improves glucose homeostasis and amplifies glucose-induced insulin secretion

**Fig. 1:**
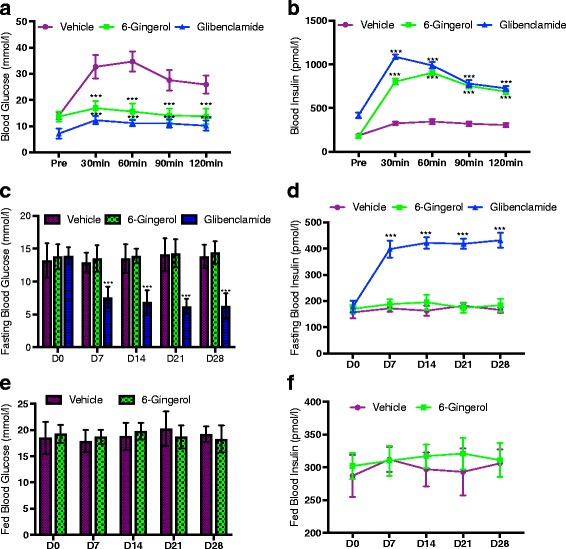
[6]-Gingerol improves glucose tolerance and homeostasis. **a** Glucose and **b** Insulin levels in 4 weeks of 200 mg/kg [6]-Gingerol treated Lepr^db/db^ mice after an oral glucose challenge of 2 g/kg. Glibenclamide treatment (0.5 mg/kg/day) was used as a positive control throughout the study period. Fasting blood **c** Glucose and **d** Insulin levels in overnight fasted mice on indicated days of the study period. Chronic effect of 6-Gingerol treatment on homeostatic blood **e** Glucose and **f** Insulin levels in non-fasted mice on indicated days of the study period. Values are presented as Mean±SD (*n* = 10 for each group). *p < 0.05, **p < 0.01, ***p < 0.001, ****p < 0.0001, n.s. not significant (Repeated measures ANOVA, Bonferroni correction)

We first evaluated the previously published glucose lowering effect of 6-Gingerol [10]. After 4-weeks of treatment, the Lepr^db/db^ mice were tested for fasting blood glucose and glucose tolerance profile. [6]-Gingerol significantly improves the glucose tolerance of the treated subjects (Fig. 1a). However, the treatment had no effect on fasting or homeostatic blood glucose level (Fig. 1c, e). As insulin controls the glucose homeostasis, we measured the dynamics of insulin secretion following 6-Gingerol treatment. In accordance with the glucose homeostasis profile, we observed a significant rise in plasma insulin in treated subjects only after glucose administration (Fig. 1b). [6]-Gingerol alone did not alter insulin secretion in homeostatic and/or fasting conditions (Fig. 1d, f), which justifies that 6-Gingerol has no effect on fasting or homeostatic blood glucose.

### Increased plasma GLP-1 level and activated cAMP/PKA/CREB pathway is crucial for insulin secretagogue activity of [6]-Gingerol

**Fig. 2:**
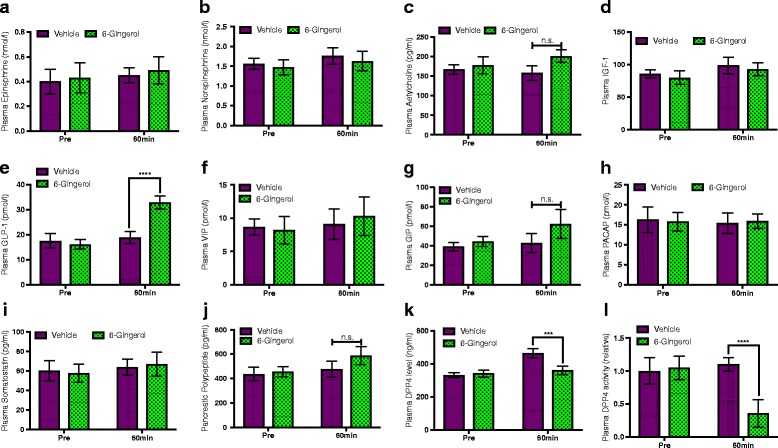
[6]-Gingerol increases plasma level of GLP-1. 4 weeks of 200 mg/kg [6]-Gingerol treated overnight fasted Lepr^db/db^ mice were challenged with a bolus of oral glucose load (2 g/kg) and plasma levels of **a** Epinephrine, **b** Norepinephrine, **c** Acetylcholine, **d** IGF-1, **e** GLP-1, **f** VIP, **g** GIP, **h** PACAP, **i** Somatostatin, **j** Pancreatic polypeptide, **k** DPP4, and **l** plasma DPP4 activity was measured before and 1 h after the glucose insult. IGF-1: Insulin-like growth factor-1; GLP-1: Glucagon-like peptide-1; VIP: Vasoactive intestinal peptide; GIP: Gastric inhibitory polypeptide; PACAP: Pituitary adenylate cyclase-activating peptide. Values are presented as Mean±SD (*n* = 10 for each group). *p < 0.05, **p < 0.01, ***p < 0.001, ****p < 0.0001, n.s. not significant (Student’s t-test)

We sought to determine the biochemical mechanism(s) behind the observed enhancement of glucose-induced insulin secretion in [6]-Gingerol treated mice. Various extracellular stimuli other than glucose could enhance or amplify insulin secretion that is triggered by glucose. Among these stimuli, different endocrine hormones, such as Acetylcholine, GIP, GLP-1 to name a few, are highly capable of exerting additive or synergistic effect on insulin secretion [27]. To determine whether [6]-Gingerol imparts its effect on insulin secretion through the hormones, we assessed the plasma levels of different endocrine hormones in treated mice after glucose feeding. Plasma GLP-1, GIP, Acetylcholine, and Pancreatic polypeptide levels were increased in the treated subjects compared to their untreated counterparts. Among these, only GLP-1 level was increased dramatically and significantly (Fig. 2a-j). Interestingly, DPP4 (dipeptidyl peptidase IV) level and activity were significantly diminished following [6]-Gingerol treatment (Fig. 2k, l), which might explain the high level of GLP-1 in the treated mice.

**Fig. 3:**
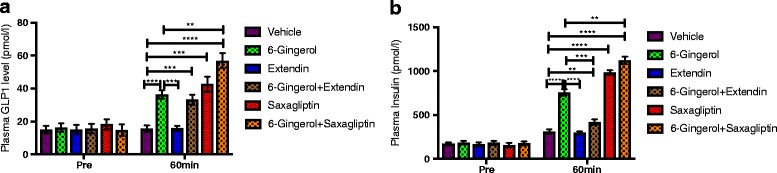
GLP-1 mediates the glucose-induced insulin secretion activity of [6]-Gingerol. Lepr^db/db^ mice treated for 4 weeks with daily 200 mg/kg [6]-Gingerol were fasted overnight and plasma **a** GLP-1 and **b** Insulin levels were measured after 1 h of an acute oral glucose challenge (2 g/kg). Different groups of the vehicle and 6]-Gingerol treated mice were additionally treated with pharmacological modulators of GLP-1: Extendin (9-39) and Saxagliptin. Exendin (9–39), a GLP-1 receptor antagonist; dose 300 pmol/kg/min; administered through a femoral vein catheter continuously for 30mins while the subjects were kept under sodium pentobarbital anesthesia. Saxagliptin, an inhibitor of dipeptidyl peptidase- 4; dose 10 μmol/l; administered orally 4 h before the glucose load. Values are presented as Mean±SD (*n* = 8 mice for each group). *p < 0.05, **p < 0.01, ***p < 0.001, ****p < 0.0001, n.s. not significant (One-way ANOVA, pair-wise comparison, Bonferroni correction)

Next, to validate whether increased GLP-1 is responsible for the [6]-Gingerol effect on insulin secretion, we employed a pharmacological approach to modulate GLP-1 level in the [6]-Gingerol treated mice and recorded the corresponding insulin levels. Saxagliptin, an inhibitor of DPP4, synergistically increased the levels of both GLP-1 and insulin in [6]-Gingerol treated mice. Likewise, Extendin (9-39), a GLP-1 receptor antagonist, treatment abolished the enhancement of insulin secretion by [6]-Gingerol (Fig. 3a and b).

**Fig. 4:**
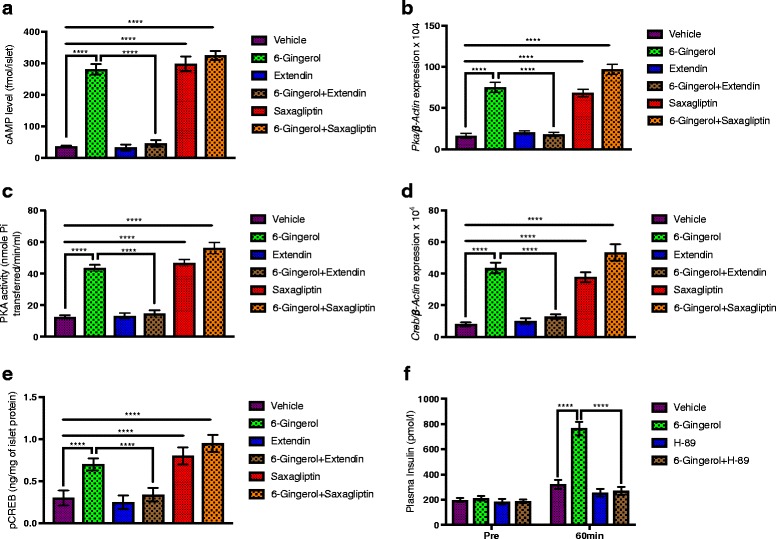
[6]-Gingerol activates cAMP/PKA/CREB pathway in pancreatic islets. Following an oral glucose load, pancreatic islets were isolated from Lepr^db/db^ mice treated with [6]-Gingerol treated (4 weeks, 200 mg/kg/day orally) with or without different pharmacological modulators of GLP-1. **a** cAMP content, **b** *Pka* expression, **c** PKA activity, **d** *Creb* expression, and **e** pCREB (S133) levels were measured in the islets isolated from different treatment groups (*n* = 8 mice for each group in **a**-**e**). **f** Plasma Insulin level following acute glucose administration (2 g/kg, oral) in [6]-Gingerol treated (4 weeks, 200 mg/kg/day orally) mice with/without 7 days’ treatment of H-89 (0.2 mg/100 g i.p.). H-89 is a selective inhibitor of cAMP-dependent protein kinase (PKA). Values are presented as Mean±SD (*n* = 8 mice for each group in **f**). *p < 0.05, **p < 0.01, ***p < 0.001, ****p < 0.0001, n.s. not significant (One-way ANOVA, pair-wise comparison, Bonferroni correction)

GLP-1 induces insulin secretion through cAMP and PKA [23]. To understand the molecular mechanism of the insulin secretagogue action of [6]-Gingerol, we assessed the status of GLP-1/cAMP/PKA pathway in pancreatic islets. Pancreatic islets were isolated from [6]-Gingerol treated mice, with or without different pharmacological agonist/antagonist co-treatments, after oral glucose administration and processed for gene expression and protein analysis. Islets from the [6]-Gingerol treated mice had very high levels of cAMP (Fig. 4a). The expression of *Pka* and *Creb*, and protein levels of PKA and activated phospho-CREB were also significantly increased (Fig. 4b-e). In the Extendin (GLP-1 receptor antagonist) co-treated mice, all the cAMP, PKA, and CREB levels in islets were reduced. Whereas, in the islets isolated from Saxagliptin (inhibitor of DPP4) co-treated mice, all these key components downstream of GLP-1 for insulin secretion were significantly increased (Fig. 4b-e). Importantly, H-89 (PKA inhibitor) co-treatment reduced plasma insulin level significantly (Fig. 4f). These results confirm that [6]-Gingerol enhances glucose-triggered insulin secretion through GLP-1, and GLP1/cAMP/PKA/CREB pathway activation is required for the said activity.

### [6]-Gingerol regulates expression of insulin granule exocytosis regulatory components *Rab27a* and *Slp4-a/Granuphilin* in pancreatic islets

**Fig. 5:**
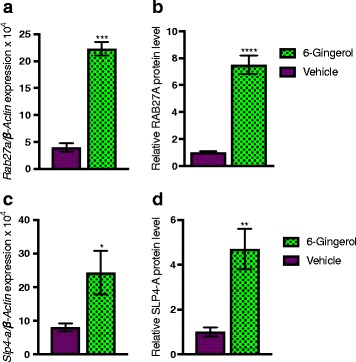
[6]-Gingerol upregulates *Rab27a* and *Slp4-a* in pancreatic islets. *Rab27a* (**a**) expression and **b** protein level, and *Slp4-a* (**c**) expression and **d** protein level in islets isolated from [6]-Gingerol treated (4 weeks, 200 mg/kg/day orally) Lepr^db/db^ mice following an oral glucose load. Values are presented as Mean±SD (*n* = 8 mice for each group). *p < 0.05, **p < 0.01, ***p < 0.001, ****p < 0.0001, n.s. not significant (Student’s t-test)

Insulin vesicle exocytosis is a major event in the glucose-stimulated insulin secretion phenomenon. The *Rab27*, a member of Rab family of small GTPases, and it’s complex partner protein *Slp4-a/ Granuphilin* is known to regulate the docking of insulin-containing dense-core vesicles to the plasma membrane for efficient exocytosis and insulin release following glucose stimulation [28, 29]. To determine whether [6]-Gingerol have any effect on insulin granule exocytosis, we assessed the expression of *Rab27a* and *Slp4-a/ Granuphilin* in the pancreatic islets. qRT-PCR analysis and ELISA confirmed that both *Rab27a* and *Slp4-a* genes expressions and protein levels were upregulated (Fig. 5a-d). Taken together, the gene expression pattern suggests that [6]-Gingerol might also regulate exocytosis of insulin-containing vesicles.

### Increased expression and membrane presentation of *Glut4* glucose transporters facilitate enhanced glycogen deposition in skeletal muscle following [6]-Gingerol treatment

**Fig. 6:**
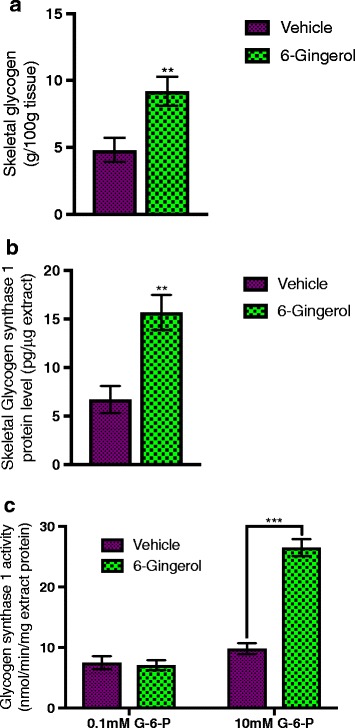
[6]-Gingerol increases skeletal glycogen deposition. **a** Total skeletal muscle glycogen, **b** skeletal Glycogen synthase 1 protein level, and **c** Glycogen synthase 1 activity were measured from the skeletal smooth muscle collected from the [6]-Gingerol treated (4 weeks, 200 mg/kg/day orally) Lepr^db/db^ mice following an oral glucose load (2 g/kg). Values are presented as Mean±SD (*n* = 10 for each group). *p < 0.05, **p < 0.01, ***p < 0.001, ****p < 0.0001, n.s. not significant (Student’s t-test)

Insulin released following food intake signals to the liver for reduced gluconeogenesis, while simultaneously increases glucose clearance from the blood by means of glycogenesis in the skeletal muscle and adipose tissue [30]. To further characterize the anti-diabetic potential of [6]-Gingerol, we evaluated the glycogenesis dynamics in treated mice following glucose intake. [6]-Gingerol treatment substantially increased the amount of deposited glycogen in the skeletal muscle (Fig. 6a). Glycogen synthase 1, which converts the excess glucose to glycogen in skeletal muscle [31], expression and activity were significantly upregulated in the muscle of the treated mice (Fig. 6b, c).

**Fig. 7:**
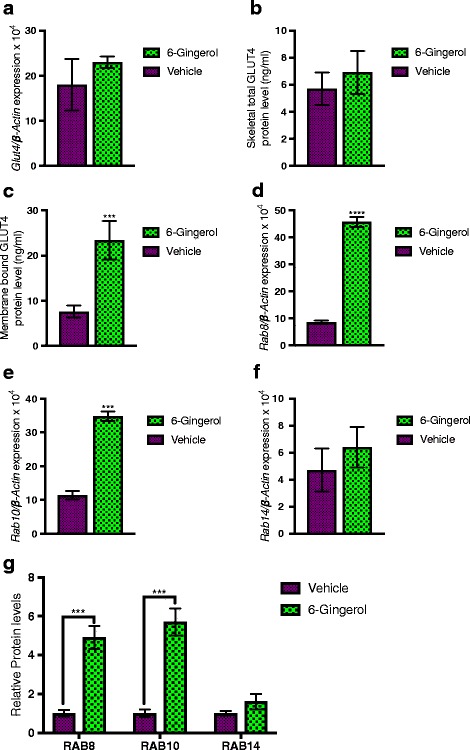
[6]-Gingerol increases membrane GLUT4 presentation by upregulating *Rab8* and *Rab10* expression. Skeletal smooth muscle and the membrane fraction of the skeletal smooth muscle were isolated by following processes described in the materials and methods section. **a** *Glut4* expression, **b** skeletal total GLUT4 protein level, and **c** GLUT4 protein level in membrane fraction was analyzed in [6]-Gingerol (4 weeks, 200 mg/kg/day orally) and vehicle treated mice. Expression of **d** *Rab8*, **e** *Rab10*, and **f** *Rab14* in the skeletal muscle was assessed by qRT-PCR analysis. The abundance of **g** RAB8, RAB10, and RAB14 proteins in skeletal muscle homogenate isolated from different treatment groups were measured by ELISA. Values are presented as Mean±SD (*n* = 10 for each group). *p < 0.05, **p < 0.01, ***p < 0.001, ****p < 0.0001, n.s. not significant (Student’s t-test)

Glucose transporters are membrane proteins that regulate the glucose transport through any membrane. GLUT4, a member of GLUT family of glucose transporters, facilitates the transport of glucose in the skeletal muscle and adipose tissue [32]. We hypothesized that the observed increased amount of glycogen in the skeletal muscle is, at least partially, due to increased glucose uptake by the GLUT4 transporters. Unexpectedly, we did not see any change in the gene expression of *Glut4* or total cellular GLUT4 content in the skeletal muscle of the treated mice compared to the untreated mice (Fig. 7a, b). However, we found that the membrane fraction the skeletal muscle was highly enriched for GLUT4 (Fig. 7c). This led us to change our initial assumption that [6]-Gingerol increases *Glut4* expression. In lieu to the increased expression, this data suggested that [6]-Gingerol increases GLUT4 membrane presence. Three members of Rab family of GTPases, RAB8, RAB10, and RAB14, were previously reported to regulate the membrane fusion of GLUT4 containing endosomal vesicles [33, 34]. Next, we investigated whether the GLUT4 enrichment of the membrane fraction was because of these Rab GTPases. Importantly, *Rab8* and *Rab10* expression and protein content were significantly increased in the [6]-Gingerol treated mice (Fig. 7d-g). Collectively, these results suggest that [6]-Gingerol increases glucose uptake in skeletal muscle through increased membrane docking of GLU4 by upregulating *Rab8* and *Rab10*, and promotes glycogenesis through increased Glycogen synthase 1 expression and activity.

## Discussion

In this current study, we aimed to characterize the mechanism of action of anti-hyperglycemic activity of [6]-Gingerol. Here, we have found that [6]-Gingerol potentiates glucose-stimulated insulin secretion through GLP-1 mediated pathway. Additionally, our data also suggest that 6-Gingerol increases insulin exocytosis and enhances glucose utilization in skeletal muscle.

[6]-Gingerol, a major constituent of ginger, was previously reported to have anti-hyperglycemic activity [10]. To understand the underlying mechanism, we utilized Lepr^db/db^ mice as type 2 diabetic model animal and subjected them to 4-weeks of daily oral administration of [6]-Gingerol. We found that unlike many antidiabetic agents, [6]-Gingerol does not induce fasting hypoglycemia, rather it increases blood insulin and reduces blood glucose level only after food intake. This could be therapeutically very potential because drug-induced hypoglycemia is a big concern in the treatment of the diabetic patients [35, 36].

Upon digestion of the food, glucose enters the blood stream and increases the blood glucose level. In response to this increase, insulin is secreted from the pancreatic beta cells. Secreted insulin increases peripheral glucose utilization and decreases hepatic gluconeogenesis. These set of events restore the blood glucose level to the physiologic range within 2–3 h after the meal. This process fails to maintain glucose homeostasis in type 2 diabetic condition, where both the insulin secretion and glucose utilization mechanisms are dysfunctional [32, 13].

Glucose-induced insulin secretion could be amplified by various extracellular stimuli such as gastrointestinal hormones [27]. Accordingly, we measured the plasma levels of different circulating gastrointestinal hormones and found that GLP-1 is increased after [6]-Gingerol treatment. By modulating the GLP-1 level pharmacologically, we confirmed that GLP-1 is responsible for the insulin secretagogue activity of [6]-Gingerol. This observation is very significant because GLP-1 has long been known to enhance the glucose-stimulated insulin secretion through so-called “incretin effect” [37]. An intravenous infusion of GLP-1 could decrease blood glucose level in type 2 diabetic patients [38]. GLP-1 mimetic drugs or DPP4 inhibitors that increase plasma GLP-1 level have already shown huge potential to treat type 2 diabetes [39]. Mechanistically, GLP-1 binds to the GLP-1 receptor on the pancreatic beta cells and increases cyclic adenosine monophosphate (cAMP), which consequently activates Protein Kinase A (PKA) and leads to insulin vesicle exocytosis [23]. Here, we have found that [6]-Gingerol activates GLP-1/cAMP/PKA pathway and this pathway is essential for the observed enhanced insulin secretion activity of [6]-Gingerol.

Insulin is stored in large dense core vesicles in the pancreatic β-cells and secreted by exocytosis in response to nutrient stimuli and different hormonal modulators [40]. In this tightly regulated exocytosis process, which is a determining step in achieving high blood insulin level following food intake to maintain glucose homeostasis, secretory vesicles fuse with the cell membrane to release insulin to the extracellular space [41]. Rab GTPases, a large family of small GTPases, regulate membrane identity and vesicle budding, motility, and fusion [42]. Among others, *Rab27a* regulates the exocytosis of insulin-containing dense-core granules through the effector protein *Slp4-a* [28]. Interestingly, we have found that [6]-Gingerol increases both the *Rab27a* and its effector protein *Slp4-a* in the pancreatic islets, which suggest that [6]-Gingerol could enhance insulin secretion by facilitating the insulin containing vesicle exocytosis.

Insulin promotes the storage and synthesis of macromolecules and inhibits their catabolism and release into the blood [43]. Approximately 80% of total glucose clearance in homeostatic condition is handled by skeletal muscle, with the rest by adipose and other insulin-sensitive tissues [44]. Regulated transportation of glucose into the cells, primarily skeletal myocytes and adipocytes, mediated by the facilitative glucose transporter GLUT4, is one of the key processes of the peripheral glucose clearance mechanism [45]. Intriguingly, [6]-Gingerol was previously reported to regulate glucose metabolism through AMPK mediated pathway [16]. To further determine the anti-hyperglycemic potential of 6-Gingerol, we assessed the effect of [6]-Gingerol on peripheral glucose deposition. We found that [6]-Gingerol increased the muscular glycogen deposition, possibly through upregulated Glycogen synthase 1 expression and activity. Unexpectedly, we did not see any increase in total GLUT4 protein level or gene expression in skeletal muscle. Importantly, we found that membrane fraction of skeletal myocytes was highly enriched with GLUT4. It is known that secreted insulin binds to the insulin receptor on skeletal myocytes and triggers intracellular signaling, which in turn stimulates translocation of the GLUT4 vesicle to the plasma membrane [46]. Our data suggest that [6]-Gingerol possibly increases GLUT4 containing vesicle membrane docking. Not to surprise, *Rab8* and *Rab10*, which are among the major regulators of GLUT4 vesicle exocytosis [34], were increased in the skeletal muscles after [6]-Gingerol treatment. Taken together, 6-Gingerol enhances glucose utilization in skeletal muscle through increased membrane presentation of GLUT4.

## Conclusion

Collectively, the present study has demonstrated that [6]-Gingerol enhances glucose-stimulated insulin secretion by activating GLP-1 mediated insulin secretion pathway and regulating insulin granule exocytosis, and increases glucose uptake in skeletal muscle by increasing GLUT4 membrane expression. How 6-Gingerol increases plasma GLP-1 level to exerts its observed insulin secretagogue activity is yet to be answered. One possible mechanism could be increasing GLP-1 plasma half-life by counter-acting dipeptidyl peptidase-4 (DPP4), which is known to cleave and in turn inactivate GLP-1 [47]. Intriguingly, we found decreased level of plasma DPP4 in [6]-Gingerol treated subjects. In our future studies, we hope to thoroughly investigate the mechanisms by which [6]-Gingerol regulates GLP-1 to ameliorate hyperglycemia.

## Additional file

Additional file 1: Table S1. Primers used in the study. (DOCX 10 kb)
