# Supplementary material for: [6]-Gingerol, from Zingiber officinale, potentiates GLP-1 mediated glucose-stimulated insulin secretion pathway in pancreatic β-cells and increases RAB8/RAB10-regulated membrane presentation of GLUT4 transporters in skeletal muscle to improve hyperglycemia in Leprdb/db type 2 diabetic mice
Source: BMC Complement Altern Med. 2017 Aug 9;17:395. doi: 10.1186/s12906-017-1903-0 (PMC5550996; doi:10.1186/s12906-017-1903-0)
Supplement: Additional file 1: Table S1. — Primers used in the study. (DOCX 10 kb) [file 12906_2017_1903_MOESM1_ESM.docx]

| Table S1: Primers used in the study | | |
| --- | --- | --- |
| Gene name | Forward primer | Reverse primer |
| *Pka* | AGATCGTCCTGACCTTTGAGT | GGCAAAACCGAAGTCTGTCAC |
| *Creb* | AGCAGCTCATGCAACATCATC | AGTCCTTACAGGAAGACTGAACT |
| *Rab27a* | TCGGATGGAGATTACGATTACCT | TTTTCCCTGAAATCAATGCCCA |
| *Slp4-1* | TGAGAAAAGGATTCGGCGACT | CTGGCACAGGTTCGATCACT |
| *Glut4* | GTGACTGGAACACTGGTCCTA | CCAGCCACGTTGCATTGTAG |
| *Rab8a* | GTAGGGAAGACCTGTGTCCTG | CGTGATTGTCCGAAACCGC |
| *Rab10* | GGCAAGACCTGCGTCCTTTT | GTGATGGTGTGAAATCGCTCC |
| *Rab14* | ATGGCAACTGCACCGTACAA | CTCCGTGTAACCGCTCTGA |
| *B-actin* | GGCTGTATTCCCCTCCATCG | CCAGTTGGTAACAATGCCATGT |
